# Hidden face of pandemic – Case study of an art therapy process during the pandemic of the virus COVID-19

**DOI:** 10.1192/j.eurpsy.2023.806

**Published:** 2023-07-19

**Authors:** I. Barun, D. Svetinović, S. Vuk Pisk, V. Grošić, I. Filipčić

**Affiliations:** University psychiatric hospital Sveti Ivan, Zagreb, Croatia

## Abstract

**Introduction:**

Pandemic caused by the virus COVID-19 had a significant impact on mental health of the population, not only by increasing the levels of stress and anxiety, but by affecting the most vulnerable ones, aggravating the symptoms of mental illnesses in people suffering from one of the mental health conditions [1], including the people suffering from schizophrenia. Pandemic made the increased need of that particular patient population for various psychotherapeutic and sociotherapeutic interventions even more evident. Art therapy is a form of psychotherapy that in itself integrates expressive characteristics of art with explorative characteristic of psychotherapy using the visual language of arts as the main media of communication and expression. Art therapy has been used from its beginnings with people suffering from one of the psychotic disorders [2] and it is enlisted today in NICE guidelines as one of the psychological therapies of schizophrenia [3].

**Objectives:**

To understand and to activate the potential of artistic expression in patients suffering from psychotic disorders during the pandemic of virus COVID-19.

**Methods:**

During the period of lockdown in pandemic of virus COVID-19, a young male patient suffering from schizophrenia was admitted to the Acute ward of the University psychiatric hospital Sveti Ivan in Zagreb. As the patient was keen on visually expressing himself, five individual psychodynamically oriented art therapy sessions were carried out on a weekly basis with professionally trained art therapist during the period of patient’s hospitalization. The patient was offered various art materials allowing him to visually express himself in a free manner and the artistic artefact created during the process served as a catalyst for later therapeutic work.

**Results:**

During the therapeutic process, single image was being gradually made and developed session by session. As new layers of color and form were added to the painting, each session revealed new layers of meaning and symbolism to both patient and therapist. First sessions pertained to the anxiety caused by the experience of pandemic, but as the process moved forward, deeper subject matters were brought to the surface, such as the nature of the therapeutic relationship, patient’s *splitting*, hidden aggressive potentials and, in the end, the nature of father-son relationship connecting the image of coronavirus causing fear and discomfort with the image of the oppressive father.

**Conclusions:**

Circumstances caused by the pandemic of virus COVID-19 aggravated the patient’s symptoms and his internal conflicts. The art therapeutic process, with its possibility of projections and its multilayered interpretations, enabled the patient to express the true conflict and disturbing content hiding underneath the anxiety related to the pandemic of coronavirus which the patient was primarily complaining about.

**Disclosure of Interest:**

None Declared

